# Structure and Behavior of Human α-Thrombin upon Ligand Recognition: Thermodynamic and Molecular Dynamics Studies

**DOI:** 10.1371/journal.pone.0024735

**Published:** 2011-09-14

**Authors:** Vivian de Almeira Silva, Maria Thereza Cargnelutti, Guilherme M. Giesel, Leonardo C. Palmieri, Robson Q. Monteiro, Hugo Verli, Luis Mauricio T. R. Lima

**Affiliations:** 1 School of Pharmacy, Federal University of Rio de Janeiro (UFRJ), Rio de Janeiro, Brazil; 2 Federal Institute of Rio de Janeiro for Science and Technology Education, Rio de Janeiro, Brazil; 3 Medical Biochemistry Institute, Federal University of Rio de Janeiro (UFRJ), Rio de Janeiro, Brazil; 4 School of Pharmacy, Federal University of Rio Grande do Sul, Porto Alegre, Brazil; 5 Center for Biotechnology, Federal University of Rio Grande do Sul, Porto Alegre, Brazil; University of Akron, United States of America

## Abstract

Thrombin is a serine proteinase that plays a fundamental role in coagulation. In this study, we address the effects of ligand site recognition by alpha-thrombin on conformation and energetics in solution. Active site occupation induces large changes in secondary structure content in thrombin as shown by circular dichroism. Thrombin-D-Phe-Pro-Arg-chloromethyl ketone (PPACK) exhibits enhanced equilibrium and kinetic stability compared to free thrombin, whose difference is rooted in the unfolding step. Small-angle X-ray scattering (SAXS) measurements in solution reveal an overall similarity in the molecular envelope of thrombin and thrombin-PPACK, which differs from the crystal structure of thrombin. Molecular dynamics simulations performed with thrombin lead to different conformations than the one observed in the crystal structure. These data shed light on the diversity of thrombin conformers not previously observed in crystal structures with distinguished catalytic and conformational behaviors, which might have direct implications on novel strategies to design direct thrombin inhibitors.

## Introduction

Thrombin is a serine proteinase that plays a fundamental role in several important physiological and pathological processes, such as the coagulation, anticoagulation and fibrinolytic pathways, and is therefore becoming an important target for drug design. Alpha-thrombin (αTh) is composed of two disulfide-linked chains, L (“light”) and H (“heavy”), with the catalytic site located in chain H. Understanding the molecular basis of ligand recognition at the active site is key for developing new compounds as candidates for drugs that aim to intervene in coagulation.

The crystal structure of αTh was elucidated more than 20 years ago [1]; since then more than 300 crystal structures have been reported so far, including complexes with various ligands, structures solved under varying chemical conditions such as pH, precipitants and coadjuvants, mutants designed to abrogate the autoproteolytic degradation [2], [3] and the wild-type recombinant human αTh in the absence of Na^+^ ions [4]. From this large database, no large conformational changes are observed [5]. Only limited overall changes with global deviations on the order of about 1 Å and some limited local shifts in loops of small secondary structure elements are seen. Despite the wealth of structures available to date, there is no consolidated structural study in solution of wild-type human αTh in the absence of inhibitors.

αTh can be reversibly unfolded by guanidine hydrochloride (GdmCl), urea and high hydrostatic pressure, with disruption of its catalytic site [6]–[8]. However, both the thermodynamics and structural consequences of ligand binding to the active site remain elusive. In the present work, we performed a comparative analysis between αTh in the free form and in the bound state in solution. To address this issue we used D-Phe-Pro-Arg-chloromethyl ketone (PPACK), a selective αTh inhibitor [9], which has long been used as a template in designing drugs that target αTh [5]. We have combined small-angle X-ray scattering, molecular dynamics simulations and equilibrium and kinetic folding thermodynamic measurements to dissect the energetics and molecular features of αTh and αTh-PPACK. Evidence for dynamic and conformational changes between these forms is provided here, as well as the mapping of a conformer in the equilibrium and kinetic folding pathway, which correlates with increased αTh activity.

## Materials and Methods

### Material

Distilled water was deionized to less then 1.0 µS and filtered through a 0.22 µm-pore membrane in a water purification system prior to use. Hen egg white lysozyme (HEWL) and bovine serum albumin (BSA) were purchased from Sigma (Sigma-Aldrich Chem. Co, Saint Louis, Il). S-2238 was obtained from Chromogenix (Mölndal, Sweden); D-Phe-Pro-Arg-cloromethylketone (PPACK) was purchased from Calbiochem. Human αTh was purified as previously described [10]. Protein concentration was determined by UV absorbance at 280 nm [11], [12]. Guanidine hydrochloride (GdmCl) stock solutions were prepared immediately before use, and the concentration was verified as described previously [13], [14]. All other reagents were of analytical grade. All buffers and solutions were prepared immediately prior use.

### Small angle X-ray scattering

Small-angle X-ray scattering (SAXS) experiments were carried out at the SAS1 and SAS2 beam lines [15] at the LNLS (National Synchrotron Light Laboratory, Campinas, SP, Brazil), with either 50 or 100 µM αTh in either the free or inhibited form (1.2 molar excess of PPACK). All samples were prepared in 20 mM Tris.HCl, 100 mM NaCl, 15 mM EGTA, 10 mM MES, pH 6.0 at 25°C, centrifuged for 15 min at 15,000 g at 4°C and maintained on ice until data collection. No proteolysis was observed as judged by SDS-PAGE after SAXS measurements (data not shown). Monodispersity was confirmed by dynamic light scattering measurements (not shown).

SAXS data were performed in duplicate with equivalent results using a one-dimensional position-sensitive detector (PSD 1D Hecus; SAS1 beamline) and a bidimensional detector (MarCCD345) at the SAS2 beamline, on different occasions. The wavelength (λ) was set at 1.488 Å; the sample-detector distance was set to provide a useful *q*-range from 0.02 Å^−1^ to 0.30 Å^−1^, calculated according to the following equation:
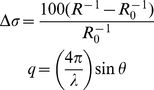
(1)where *q* is the modulus of the scattering vector, and 2θ is the scattering angle. The scattering curves of the protein solutions and buffers were collected in several successive frames of 900 s each to monitor for radiation-induced protein oxidation. The data-reduction routine included normalization of the one-dimension scattered data to the intensity of the transmitted incident beam, correction for the detector response, incident beam intensity, sample absorption, and blank and detector background subtraction. Measurements were performed using at least three different protein batches.

Guinier analysis [16] was applied to further evaluate monodispersity of the samples and to determine the radius of gyration (*R_g_*) of αTh and αTh-PPACK. The *R*_g_ and the scattered intensity, I_0_(q), were inferred, respectively, from the slope and the intercept of the linear fit of ln[*I(q)*] versus *q* ^2^ in the *q*-range q*R_g_<1.3 [16]. The same parameter was also obtained from the data fit of the merged curve by the indirect Fourier transform program *Gnom* [17], [18], which also evaluates the distance-distribution function, *p(r)*. The maximum dimension, *D_max_*, was estimated from the distance distribution function *p(r)*, with the limiting distance r where *p(r)* first converges down to zero. The lack of the dependence of the structural parameters obtained by SAXS on the protein concentration indicates the absence of protein interactions at this concentration range. The oligomeric states of αTh and αTh-PPACK in SAXS measurements was confirmed from the extrapolated values of scattering intensity at zero scattering angle (I_0_) and normalized by sample concentration *C* according to *I*_0_/*C* using BSA and HEWL as reference standards [19]–[21]. The SAXS data were analyzed by fitting the theoretical scattering intensities computed from the crystal structure of PPACK-bound αTh (PDB ID 1PPB) using *Crysol* [22], taking into account the influence of the hydration shell. Similar results were obtained for measurements performed either at 50 αM or 100 µM of αTh and αTh-PPACK.

### Equilibrium unfolding and refolding

Equilibrium unfolding was performed by incubating 500 nM αTh or αTh-PPACK in the indicated GdmCl concentration and allowing the reaction to equilibrate for at least 60 min, which is sufficient time to achieve equilibrium [8]. Reversibility experiments were performed by incubating αTh or αTh-PPACK at 20 µM in 4.5 M GdmCl, diluting it to 500 nM supplemented with the necessary amount of GdmCl to achieve the indicated concentration, and measuring fluorescence emission. Fluorescence measurements were performed separately with three protein batches in three spectrofluorimeters, a Jasco FP-6300 (Jasco Inc, USA) and two Cary Eclipse (Varian Inc), with excitation set to 280 nm and emission scanned from 300 to 420 nm at a scanning rate of 100 nm/min and response 8 seconds, datapitch 0.5 nm and excitation and emission slits 2.5 nm. Fluorescence spectra were quantified by the center of spectral mass <v> according to Eq [2]):
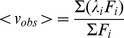
(2)where F_i_ is the fluorescence emitted at wavelength λ_i_ and the summation is carried out over the range cited above. All experiments were performed at 25°C in PBS buffer (150 mM NaCl, 3 mM KCl, 2 mM KH_2_PO_4_, 10 mM Na_2_HPO_4_, pH 7.4).

Because αTh unfolding is fully reversible with no detectable hysteresis [6], [8], the transition between the native (N) and denatured (D) conformational states can be defined by an equilibrium denaturation constant, *k_den_*, as:
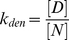
(3)and the free energy of unfolding can be calculated from
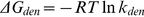
(4)where R and T are respectively the universal gas constant (1.9872 cal.mol^−1^.K^−1^) and temperature (in Kelvin).

The unfolding energy can generally be accessed from the dependence of the monitoring signal (in this case, spectral center of mass of intrinsic fluorescence emission) on the denaturant concentration, by using the linear extrapolation method [23], [24]

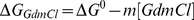
(5)where Δ*G_GdmCl_* is the free energy of unfolding at each respective GdmCl concentration, Δ*G^o^* is the standard unfolding free energy (in this case, in the absence of denaturant) and *m* is a parameter directly related to the difference in accessible surface area (ASA) between the folded and unfolded states [25]. Combination of Eq [4] and Eq [5] gives:
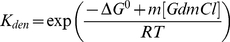
(6)Considering
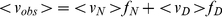
(7)and
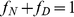
(8)where f_N_ and f_D_ are the fractional amount of protein in the native and denatured states, respectively, it follows that
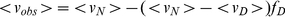
(9)where *obs*, *N* and *D* represent *observed* (i.e., at each corresponding GdmCl concentration), *native* and *denatured*. The fraction of denatured protein can be described as
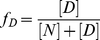
(10)Combination of Eq 3 and 10 gives
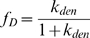
(11)and combining Eq [6], Eq [7] and Eq [10] results in
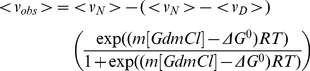
(12)which was used to fit the GdmCl-induced denaturation isotherms.

### Thermal denaturation

Experiments were performed with αTh or αTh-PPACK in PBS buffer, using both a Jasco J-715 (Jasco Corporation, Tokyo, Japan) and a Chirascan (Applied Photophysics, UK) spectropolarimeter. Heat denaturation curves were generated by monitoring the ellipticity at 222 nm. Similar transitions were obtained with a heating rate of 1 or 2°C/min, varying protein concentration and cell path length, with equivalent mean transition temperature. Temperature was varied by a Peltier controller. All experiments were performed in triplicate, with three protein batches. Unfolding curves were fit to a four-parameter sigmoid logistic function to estimate the mean transition temperature of thermal denaturation.

### Kinetic measurements

Kinetic unfolding experiments were performed using a SX18MV stopped-flow apparatus (Applied Photophysics, UK). All experiments were performed in PBS, at 25°C±0.2°C (unless otherwise stated), using syringes of different sizes for a 1∶10 mixture and final protein concentration of 900 nM. Intrinsic fluorescence was followed by setting the excitation wavelength to 280 nm, and monitoring the emission through a cut-off filter (WG320, with 50% transmitance at 320 nm). All data presented are an average of at least four runs, and the reported GdmCl concentrations are the final values under measurement. Data were adjusted to obtain the rates using non-linear least squares fitting as provided by the manufacturer, with a single exponential decay function. Kinetic refolding experiments were performed by incubating αTh or αTh-PPACK with the desired GdmCl concentration for at least 1 h and diluting the reaction 10 times with buffer and varying the amounts of GdmCl to provide the indicated final concentration of GdmCl.

### Molecular dynamic simulations

The MD simulations were performed with the αTh-PPACK complex and free αTh based on the protein structure under PDB entry 1PPB [1]. The GROMACS simulations suite [26] and GROMOS96 force field [27] were used, employing an MD protocol based on previous studies [28], [29]. The architecture of αTh-PPACK linking was made using charges, bonds, angles and dihedrals parameters founded in GROMOS96 43a1 force field. The final complex is a hemiketal tetrahedrical structure binding Ser195 and His57 side chains of the enzyme to Arg3 carbonyl carbon of the PPACK. Both αTh and the αTh-PPACK complex were solvated in rectangular boxes using a SPC water model [30] by a layer of at least 9 Å from the solute atoms. Counter ions (Cl^−^) were added to neutralize the system charges. The Lincs method [31] was applied to constrain covalent bond lengths, allowing an integration step of 2 fs after an initial energy minimization using steepest descents algorithm under periodic boundary conditions. The systems obtained, composed of αTh–solvent–ions and αTh–PPACK-solvent–ions were heated slowly from 10 to 343 Kelvin, in steps of 5 ps, in which the reference temperature was increased by 50 K. Both systems were kept at a temperature of 343 K for the rest of the trajectory. Temperature and pressure were kept constant by coupling protein, PPACK, ions, and solvent to external temperature and pressure baths with coupling constants of τ = 0.1 and 0.5 ps [32], respectively. No restraints were applied after the thermalization phase. The electrostatic interactions were evaluated by the particle–mesh Ewald method [33] with a charge grid spacing of 1.2 Å, while Coulomb and Lennard–Jones interactions were evaluated using a 9.0 Å atom-based cutoff [34]. The analyses were performed in all trajectory length, with average values of interaction energies calculated in the last 20 ns of the simulations, which last for a total of 50 ns including all previous steps. Throughout the text we use the sequence nomenclature as described elsewhere [1].

## Results

### Small-angle X-ray scattering

Ligand interaction with proteins might result in more complex structural changes beyond local interaction. To characterize the overall structural parameters that could be affected upon ligand binding to αTh, we performed small-angle X-ray scattering (SAXS). SAXS can assist the evaluation of structural patterns of proteins such as protein oligomerization and structural remodeling, and allow for a comparative analysis between the crystal and solution structure of a protein [35].

**Figure 1:**
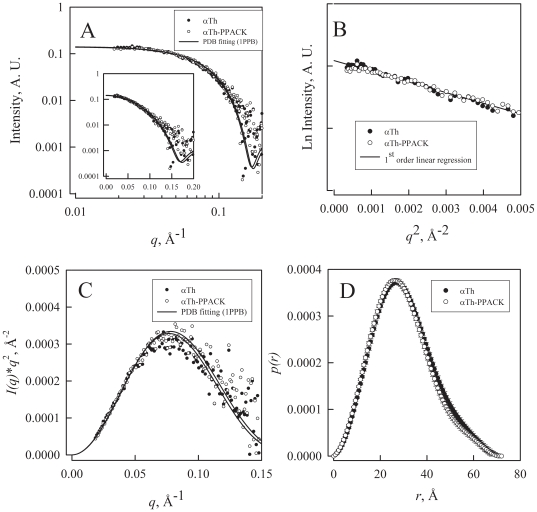
Small-angle X-ray scattering analysis of αTh and αT-PPACK. SAXS measurements of free (closed circles) and active site-bound (open symbols) αTh (**A**) Experimental scattering curves of αTh and αTh-PPACK; solid lines correspond to fits to data with the crystal structure of αTh-PPACK (1PPB) using Crysol. *Inset*: linear *q* scale. (**B**) Guinier plot of the scattering function; solid lines correspond to first-order linear regression of the data. The linearity of the Guinier plot indicated that both samples are monodispersed and constitute a unique species; (**C**) Kratky plot from raw data and fitting from panel (A); (**D**) Distance distribution functions. Details in the *Experimental Methods* section.

The X-ray scattering patterns of αTh and αTh-PPACK are very similar (Fig. 1A), with no detectable changes within the resolution limits (Fig. 1A). A Guinier analysis of SAXS data [16] provides the radius of gyration, which is similar for both αTh and αTh-PPACK, of about 23 Å (Fig. 1B; Table 1). The protein globularity is evidenced by a Kratky plot [16], [36], which also reveals that both αTh forms display no flexible and/or unfolded domains, as indicated by the typical hyperbolic distribution (Fig. 1C). Pair distribution analysis shows behavior typical of a globular protein for both αTh and αT-PPACK, confirming the similarity in radius of gyration (*R_g_* of about 23 Å) and maximum distance *D_max_* (72 Å) (Fig. 1D; Table 1).

**Table 1:** Structural and thermodynamic parameters of αTh and αTh-PPACK.

Structural Parameters^1^	αTh	αTh -PPACK
I(0)	0.150	0.147
*R_g_*, Å (Guinier)	23.4 (r^2^ = 0.978)	21.9 (r^2^ = 0.974)
*R_g_*, Å (real space; *Gnom*)	23.11±0.149	22.50±0.190
*R_g_*, Å (1PPB, *Crysol*)	23.3	21.9
*D_max_*, Å (*Gnom*)	72±2	72±2
Discrepancy value χ^2^ (*Crysol*; 1PPB.pdb)	1.67	1.54
Resolution (Å)^2^	31	31
**Thermodynamic Parameters**		
Δ*G* ^0^, kcal/mol^3^	3.68±0.26	15.33±0.88
*m*, kcal/mol.M^3^	3.12±0.21	4.96±0.28
Tm, °C^4^	58±0.2	74±0.1
ΔH^‡^, kcal/mol^5^	20.0±0.9	23.1±1.6
ΔS^‡^, kcal/mol.K^5^	0.071±0.001	0.071±0.005
ΔCp^‡^, kcal/mol.K^5^ (reference temperature = 298 K)	0.62±0.20	1.00±0.42

1 ) from SAXS measurements;

2 ) Resolution is calculated as 2π/q*_max_*;

3 ) from equilibrium GdmCl induced denaturation;

4 ) from heat induced denaturation, with equivalent results for both 1 and 2°C/min heating rate;

5 ) from kinetic measurements.

We also performed a comparative analysis of scattering data in solution for αTh and αTh-PPACK with the existing high-resolution structures by computing the theoretical scattering curves from the crystal structure of αTh-PPACK (PDB entry 1PPB) (Fig. 1, solid lines). The comparison between these simulated SAXS curves and the experimental data resulted in discrepancy values (χ^2^) of about 1.5 for both αTh and αTh-PPACK (Table 1), suggesting that the overall shapes of both αTh and αTh-PPACK are not similar to that from the crystal structure. Collectively, these data indicate that binding to PPACK does not change both the overall particle form and the molecular envelope of αTh, and that both forms have a similar overall shape in solution, which is different from that observed in thrombin crystal structures.

### Conformational changes induced by active-site occupation

**Figure 2:**
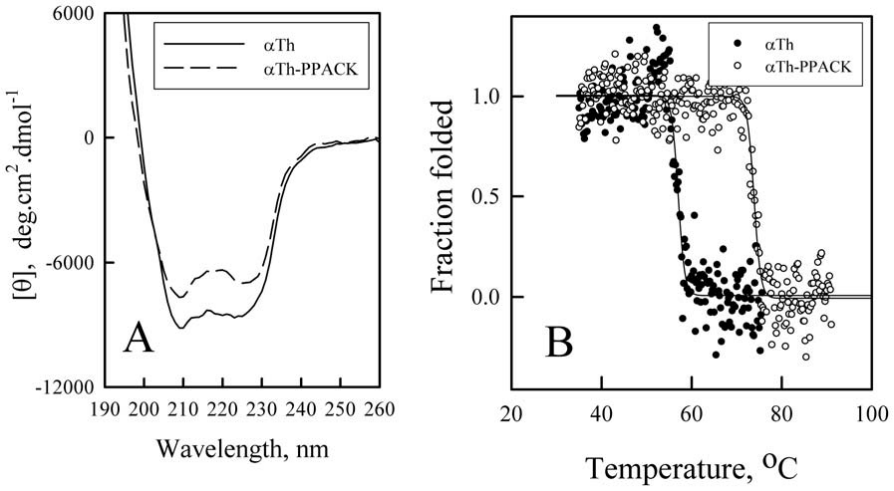
Secondary structure effects of αTh active site occupation. (**A**) CD spectra of free (continuous line) and PPACK-bound (dashed line) αTh. Spectra were collected at 30 µM for both proteins in 0.10 mm pathlength quartz cells. (**B**) Thermal unfolding of free (closed symbols) and PPACK-bound (open symbols) αTh monitored by changes in ellipticity at 222 nm throughout the process. Solid lines are linear regression. Details in the *Experimental Methods* section.

To gain more insight on the structural changes in αTh upon ligand binding to the active site, we monitored the changes in secondary structure by circular dichroism (CD). The CD spectrum of αTh is characteristic of an alpha-helix rich protein (Fig. 2A) in the far-UV region, with minima at about 222 and 212 nm. Upon binding to PPACK, the overall pattern of the αTh CD spectrum remains similar; however, a decrease in molar ellipticity takes place, indicating some degree of secondary structure rearrangements. Changes in ellipticity in the far-UV spectral region can also arise from perturbations near aromatic residues in the active site, thought the contribution from these effects is less than one order of magnitude of the protein molar ellipticity [37]. These data indicate a complex behavior of the secondary structural elements of αTh upon ligand binding.

### Thermal unfolding

To gain insight on the overall energetic contributions of PPACK binding to αTh, we used CD to monitor heat-induced denaturation of αTh by monitoring the ellipticity at 222 nm. We observed a steep decrease in ellipticity corresponding to protein unfolding transitions at 58°C and 74°C for αTh and PPACK-bound αTh, respectively (Fig. 2B). This change in thermal stability is consistent with previously-reported stabilization of bovine thrombin by PPACK [38]. The increase in stability is more likely to derive from changes in protein conformation and dynamics upon ligand binding than from its covalent linkage to thrombin or the local energetics of ligand interaction at the active site. Unfortunately, because thermal denaturation was not reversible for both αTh forms, no quantitative thermodynamic parameters could be estimated except for the apparent temperature of transition.

### Equilibrium refolding and unfolding transition

**Figure 3:**
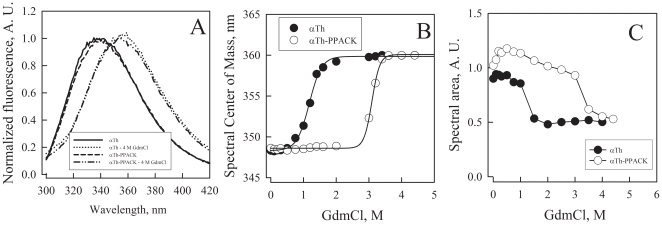
Stability effects of αTh active site occupation. Evaluation of PPACK binding to αTh was probed by equilibrium denaturation induced by GdmCl and monitored by intrinsic fluorescence spectroscopy. (**A**) Fluorescence spectra of free and PPACK-bound αTh in the presence and absence of 4 M GdmCl. (**B**) Denaturation curve of free (closed circles) and PPACK-bound (open circles) αTh was monitored by changes in the spectral center of mass. Solid lines are non-linear regression fitting with Eq [12]. (**C**) Changes in intrinsic fluorescence emission of free (closed circles) and PPACK-bound (open circles) αTh as a function of GdmCl concentration. Note the increase in fluorescence emission in a pre-transition region for αTh-PPACK, corresponding to the transition between the initial ‘native’ and the intermediate states. Excitation was set at 280 nm and emission scanned from 300 to 420 nm; both the spectral center of mass and spectral area were calculated. Details in the *Experimental Methods* section.

To further evaluate the effects of active site occupation on αTh overall stability and to estimate the energetic contribution of binding, we performed equilibrium denaturation isotherms using GdmCl as a denaturant and by monitoring changes in intrinsic fluorescence of αTh [8]. The emission spectra of both αTh forms progressively shifted to higher wavelengths, indicating the exposure of aromatic amino acids to the bulk solvent as a consequence of the unfolding process (Fig. 3A). A plot of the spectral center of mass as a function of denaturant concentration shows a sigmoid, cooperative transition with well-defined plateaus at low and high GdmCl concentration (Fig. 3B). From these data, we observe that PPACK binding to αTh induces a shift of the curve to higher denaturant concentrations, indicating an increase in protein stability (Fig. 3B).

The unfolding transition of both αTh and αTh-PPACK is completely reversible without hysteresis [6], [8]. The analysis of the changes in fluorescence intensity as a function of denaturant concentration shows an increase of about 25% for αTh-PPACK at low denaturant concentrations (below 1 M GdmCl; Fig. 3C). Such spectroscopic evidence suggests the existence of a well-populated intermediate state between the folded and unfolded states of αTh-PPACK, as previously suggested by spectroscopic and functional assays [8]. This behavior is not observed in the αTh denaturation isotherm monitored both by the spectral center of mass and fluorescence intensity, indicating either that this intermediate state does not exist during equilibrium unfolding or that it is spectroscopically silent.

Considering a reversible equilibrium system, we were able to estimate the free energy (ΔG^0^) of the transition between intermediate and unfolded species. Adjusting Eq [12] to data gives provides a ΔG^0^ of 3.7±0.3 kcal/mol for apo αTh, in excellent agreement with the unfolding ΔG^0^ of 3.4±0.3 kcal/mol obtained from our group by using urea as denaturant [8]. Adjusting Eq [12] to data provides a ΔG^0^ of 15.3±0.9 kcal/mol for αTh-PPACK. The difference between these values (ΔΔG^0^_apo-PPACK_) is the overall gain in unfolding free energy of αTh-PPACK compared to the free enzyme, which is 11.6 kcal/mol, about 3 times the free energy of unfolding for apo αTh.

From these denaturation curves we could also obtain the *m* parameter, which is directly correlated to the difference in solvent accessible surface area (ΔASA) between the folded (ASA^F^) and unfolded (ASA^U^) states [25], as follow:
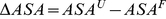
(13)


The *m* parameter also increased upon active site occupation, changing from 3.12±0.21 to 4.96±0.3 for αTh and αTh-PPACK, respectively, resulting in Δ*m* = 1.8. If PPACK binding to αTh leads to changes in solvent accessibility and thus, in conformation, we can assume that
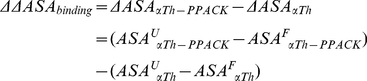
(14)


Assuming that the final unfolded states for αTh and αTh-PPACK are similar, with equivalent 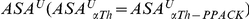, it follows that
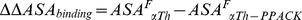
(15)


According to the GdmCl-induced unfolding curves (Fig. 2), Δ*m* is positive; therefore, ΔΔASA_binding_ would also be positive. This analysis allows us to suggest that folded αTh-PPACK has an overall conformation that is slightly less solvated and thus slightly more compact than folded, free αTh.

### Kinetic refolding and unfolding transition

To address the kinetic basis for the thermodynamic stabilization of αTh by PPACK and investigate the origins of the intermediate species, we performed fast-kinetic unfolding and refolding experiments using a stopped-flow setup by monitoring conformational changes via intrinsic fluorescence.

**Figure 4:**
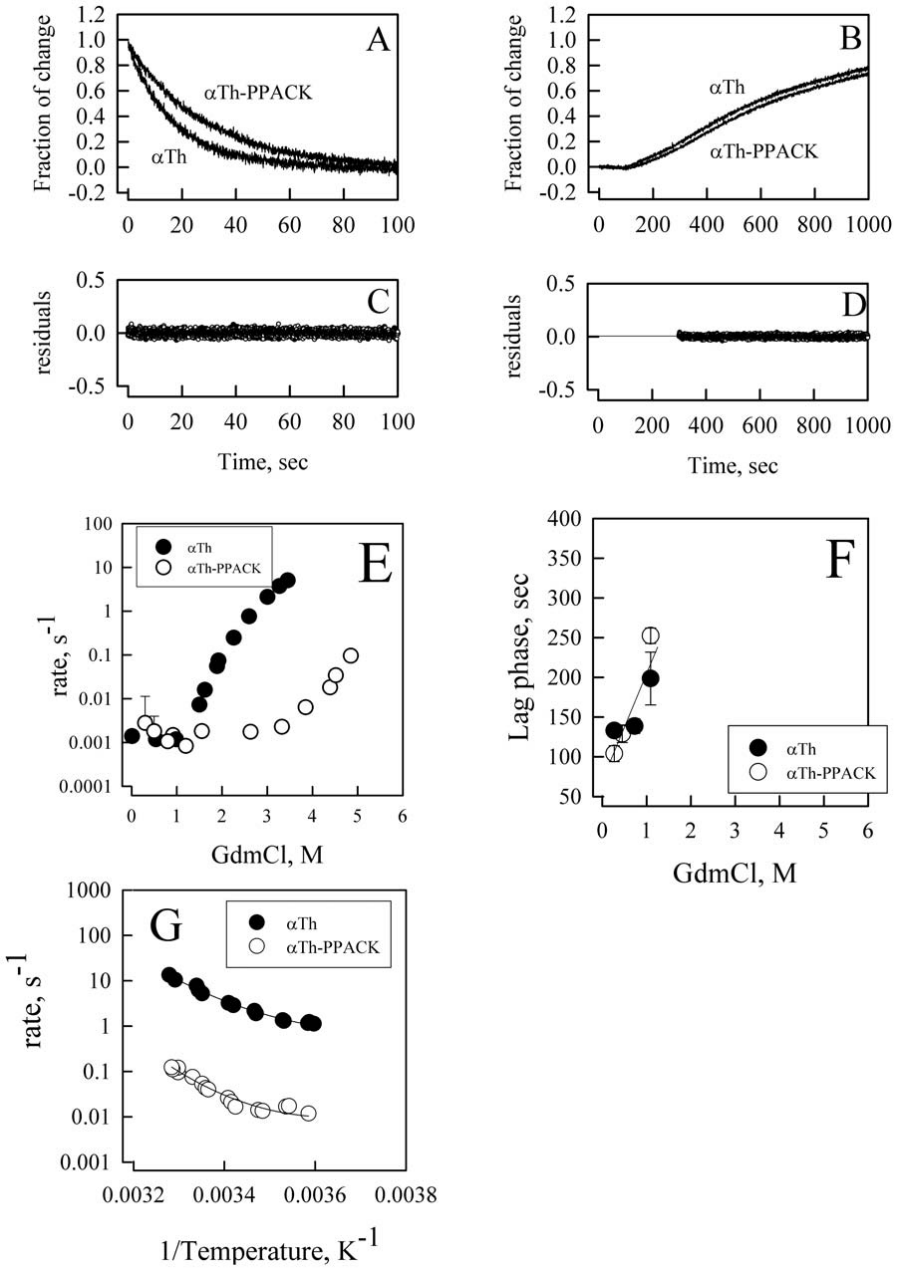
Kinetic measurements of GdmCl induced folding and unfolding transitions of αTh and αTh -PPACK. Kinetic traces (A and C) and residuals (B and D) of unfolding (A; B) and refolding (C;D) transitions of αTh (closed circles) and αTh-PPACK (open circles). The curves were fit with a single exponential decay, and the rate constant was calculated. Fitting residuals are shown for αTh (closed circles) and αTh-PPACK (open circles); (E) Chevron plot – GdmCl dependence of the apparent rate constant of folding and unfolding of αTh and αTh-PPACK. The lack of linearity in the Chevron plot indicates a kinetic mechanism more complex than a simple two-state model, and thus limits a precise quantitative analysis of the kinetic process. (F) The dependence of the refolding lag phase as a function of GdmCl. (G) Temperature dependence on the kinetics of GdmCl-induced unfolding of αTh and αTh-PPACK by jumping the GdmCl concentration from 0 to 4 M and monitoring by the changes in fluorescence intensity, as in Fig. 4A. Lines represent the best fit with Eq 22. Details in the *Experimental Methods* section.

Mixing αTh or αTh-PPACK with GdmCl at varying denaturant concentration lead to a decrease in fluorescence intensity (Fig. 4A). No further changes are observed after 10 min (data not shown). The kinetic traces for both αTh and αTh-PPACK GdmCl-induced denaturation were fit to a single exponential function according to the following equation:
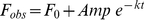
(16)where F_obs_ is the fluorescence signal measured at time t; Amp is total fluorescence change from F_0_, the initial fluorescence signal, and k is the rate constant for the observed transition. The data fit well to this equation, as indicated by the residuals (Fig. 4C). An apparent first-order kinetic event for both αTh and αTh-PPACK unfolding transitions was observed, which may indicate a simple two-state transition from folded to unfolded αTh or a multi-step process with spectroscopically silent species.

In contrast, the refolding reaction of αTh and αTh-PPACK shows a more complex behavior. The kinetic refolding for both αTh and αTh-PPACK was performed by incubating them at high GdmCl concentration and diluting back with buffer supplemented with sufficient GdmCl to provide the final desired concentration. The decrease in GdmCl concentration leads to the increase in fluorescence intensity of both αTh and αTh-PPACK which is preceded by a lag phase. These data indicate that at least two events are taking place in the course of the refolding reaction. The duration of the lag phase in the refolding kinetics is dependent on GdmCl concentration (Fig. 4F), and linear extrapolation provides a life-time of about 100 sec, corresponding to an apparent decay rate constant, k_UI_ of 0.001 sec^−1^, for the transition from the unfolded state to an intermediate state “I” which is similar for both αTh and αTh-PPACK.

The second transition in the refolding reaction can also fit well to a simple first-order kinetic function (Eq. [16]; Fig. 4B and 4D). From the analysis of the second transition in the refolding reaction, we obtain an apparent refolding kinetic rate constant *k_IN_* , which corresponds to the transition from the intermediate state “I” to the native folded “N” conformation. A plot of the observed kinetic constant *versus* the denaturant concentration is called a *Chevron* plot [39]. The linear extrapolation of the observed kinetic constant to zero concentration of GdmCl provides the *k*_UN_ and *k*_NU_. For thrombin (Fig. 4E), the apparent rate constant of refolding, *k_UN_*, converges at similar values for both αTh (*k_UN_* ^αTh^) and αTh-PPACK (*k_UN_* ^αTh-PPACK^).

Collectively, these data suggest that the mechanism of kinetic refolding is similar for αTh and αTh-PPACK, as indicated by a similar two-steps refolding. This implies that the basis for the energetic stabilization of αTh upon ligand binding is mainly determined by the kinetic unfolding pathway. Assuming that the unfolded states of αTh and αTh-PPACK are similar, it is suggestive to propose that the thermodynamic difference between αTh and αTh-PPACK relies on the native conformation and/or the activated state in the kinetic unfolding process.

In a perfect two-state conformational transition, the reference native state of a protein is a result of the balance between the refolding and unfolding kinetic constants:
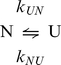
(17)where *k_UF_* and *k_FU_* are the microscopic rates of the refolding and denaturation reaction, respectively, and are related to the equilibrium denaturation constant *k_eq_* according to the following [39], [40]:
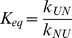
(18)


The dependence of the unfolding rate constants on GdmCl concentration deviate from linearity for αTh at concentrations higher than 2 M GdmCl (Fig. 4E). This rollover indicates that the unfolding reaction is not a simple two-step reaction, and that at least one intermediate is populated. This behavior is not observed for αTh-PPACK at the GdmCl concentrations used. Instead, a linear dependence on GdmCl concentration is observed. However, we can not rule out the existence of a denaturation intermediate for αTh-PPACK because we were not able to achieve a GdmCl concentration higher than 5 M due to limitations in the mixing procedure and the limited solubility of reagents in the stock solution. Moreover, the refolding kinetic process for both αTh and αTh-PPACK reveals two marked steps, which is direct evidence for a process involving at least three species: unfolded, intermediate and folded αTh. Therefore, based on this evidence, we may assume the existence of at least one refolding species named “I”, such that:
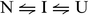
(19)


The linear dependence of the kinetic rate constant on the GdmCl concentration can be expressed by
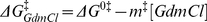
(20)where ^‡^ denotes the transition or activated state. From the kinetic refolding measurements, we observe a large dependence on *k_UI_*, the microscopic rates of the U to I transition reaction, on GdmCl concentration for both αTh and αTh-PPACK (Fig. 4F) and almost no dependence on *k_IN_*, the microscopic kinetic rates of the I to N transition reaction, on GdmCl concentration (Fig. 4E, between 0 and 1.5 M GdmCl). These data indicate that *m* ^‡^_UI_ is positive and *m* ^‡^ *_IN_* is close to zero. As previously mentioned, the *m* paramenter directly correlates to changes in solvent accessible surface area (ΔASA) between the conformers involved in the transition [25]. From this analysis, we suggest that for both αTh and αTh-PPACK the transition from αTh_Unf_ to αTh_Int_ involves change in hydration and subsequently in solvent exclusion from the surface due to protein condensation and that the transition from αTh_Int_ to αTh_Native_ is accompanied by only minor changes in the solvent accessible surface area. Moreover, these data suggest that the kinetic intermediate state in the refolding and unfolding pathway for both αTh and αTh-PPACK is equivalent to the refolding/unfolding intermediate accumulated in equilibrium, and is more closely related to the folded αTh than to unfolded αTh.

The current data clearly indicate the existence of a kinetic intermediate in the unfolding/refolding pathways for both αTh and αTh-PPACK. These data corroborate previous equilibrium measurements from our group [8]. Unfortunately, it is still not possible to confidently assign the number of refolding intermediates states or to know if each refolding intermediate is be the same in both the unfolding and refolding pathways. Instead, our data provide clear evidence for local energetic minima in the refolding pathways for both αTh and αTh-PPACK, demonstrating the diverse refolding trajectory of this enzyme.

### Activation energy for refolding kinetics

To further understand the unfolding energetic of free and PPACK-bound αTh, we performed a comparative analysis of the kinetic energetic of apo and holo αTh unfolding by measuring the thermal dependence of the denaturation kinetics of apo and holo αTh at a fixed concentration of denaturant (4 M GdmCl) by stopped-flow measurements. From the Arrhenius, it follows that
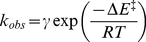
(21)where γ is a pre-exponential coefficient, T is the temperature in Kelvin, and ΔE^‡^ is the activation energy. The plot of ln *k* versus 1/RT is a linear function from which the angular coefficient corresponds to ΔE^‡^. A deviation from linearity indicates that the activation energy is dependent on temperature, and this dependence is denoted as ΔCp^‡^, the change in activation heat capacity [39], [39]–[42].

If ΔCp^‡^ contributes to the underlying process, the process can be better described according to the following:
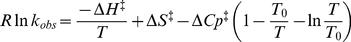
(22)where ΔH^‡^ and ΔS^‡^ are the enthalpy and entropy activation changes at the reference temperature T_0_, respectively.

From 5 to 30°C, the dependence of the apparent rate constant on temperature in the form of log*k* versus 1/T (K^−1^) is slightly non-linear (Fig. 4G). Using Eq. 22, we calculated ΔCp^‡^ of 0.62±0.20 kcal/mol.K and 1.00±0.42 kcal/mol.K for apo and holo αTh, respectively. Because the heat capacity is associated to conformational changes in the protein leading to changes in the degree of hydration of apolar groups [43], [44], we interpret the small measured ΔCp^‡^ as indicative of only a discrete difference in the accessible surface area between the folded and the unfolded active state. More importantly, the ΔCp^‡^ for apo and holo αTh are the same within error. These results indicate a similar activation mechanism in the unfolding process between apo and holo αTh and, consequently, that the large energetic differences between these species reside in the decay from activated and unfolded states.

### Molecular dynamic simulation and structural stability

We have observed thermodynamic effects of ligand binding to αTh (Fig. 2, Fig. 3 and Fig. 4). Unfortunately, there is no high-resolution structure of ligand-free, wild-type αTh, and therefore it is still not possible to gain precise insight on the structural basis of such thermodynamic behavior of αTh upon ligand binding to the active site. Due to this lack of structural information, we performed molecular dynamics simulations (MD) of both αTh and αTh-PPACK to uncover the underlying mechanism of ligand recognition and its energetic consequences.

**Figure 5:**
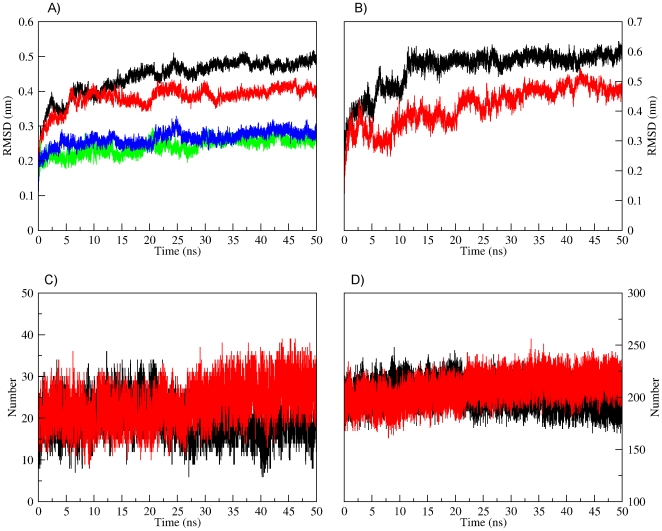
Overall properties assessed by MD simulations. RMSD values for free enzyme (black curve), αTh-PPACK (red curve), secondary structure deviation in free αTh (green curve) and in complexed αTh (blue curve), for light chain (“L”, panel **A**) and heavy chain (“H”, panel **B**); (**C**) H-bonds formed between coil-surrounded PPACK site in free αTh (black curve) and complexed form (red curve); (**D**) Total intramolecular H-bonds formed between residues for free αTh (black curve) and complexed αTh (red curve). Details in the *Experimental Methods* section.

**Figure 6:**
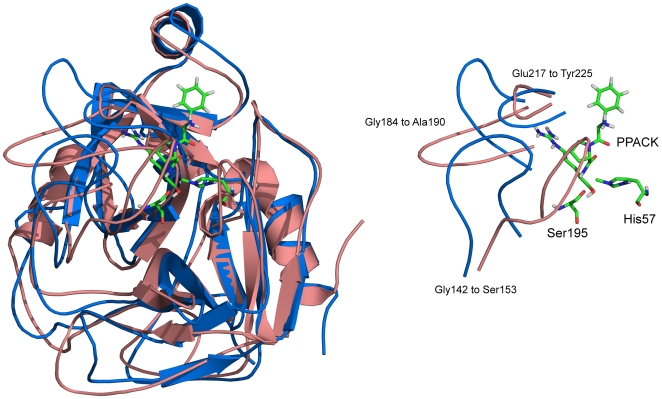
Superposition of unbounded αTh (salmon) and αTh-PPACK complex (blue) after 50 ns MD simulation. The loops surrounding the PPACK binding site in the catalytic cleft are shown in detail. Both structures deviate from the original crystallographic model used in the MD simulations (PDB ID 1PPB). Details in the *Experimental Methods* section.

We observed no meaningful differences in the radius of gyration for both αTh and αTh-PPACK throughout the simulation (data not shown), which agrees with our SAXS measurements in solution (Fig. 1) demonstrating that there are no or only minor structural changes between the two species (Fig. 1). To further monitor the conformational changes of αTh and αTh-PPACK by MD, we separately evaluated the root mean square deviation (RMSD) of the simulated systems using the crystallographic structure in L-chain (Fig. 5A) and H-chain (Fig. 5B) as a reference. Binding to PPACK resulted in increased protein rigidity with only limited change in secondary structure (Fig. 5A and B, curves in blue and green) and overall protein topology (Fig. 6A) from the initial conformation.

The change in secondary structure of αTh is accompanied to some extent by changes in the RMSD from the reference structure, which is more pronounced in the L-chain compared to the H-chain, as seen in Fig. 5A and 5B, respectively. Despite the small deviation from the initial secondary structure content we observed a high degree of fluctuation in the L-chain for both αTh and αTh-PPACK as exhibited by the large RMSD throughout the MD simulation trajectory, not only in secondary structure but also in general L-chain. In opposition, the RMSD of the H-chain occurs with only limited fluctuation throughout the simulation (Fig. 5B), which is similar for both αTh (black lines) and αTh-PPACK (red lines). Interestingly, under these conditions, there is no rigidity influence of PPACK over the αTh L-chain.

### Dominant forces participating in αTh stabilization

We can quantify the contribution of specific forces, such as intramolecular interactions within the protein and intermolecular interactions between the protein and the surrounding solvent. The three αTh loops 142–153, 184–190 and 217–225 form flaps around the active site and changes its conformation upon PPACK upon binding (Fig. 6A). In fact, these loops surrounding the PPACK binding site become less flexible upon ligand binding. This rigidity is accompanied by an increase in both hydrogen bonds between these loops in the vicinity of the active site (Fig. 5C) and in total intramolecular hydrogen bonds within αTh (Fig. 5D) Additionally, as shown in Table 2, binding to PPACK increases the interaction energy profile between residues, which is accompanied by a decrease in the interaction energy with the solvent, as evidenced by the solvation enthalpy of −17,809±431 kJ/mol for αTh, and −16,678±374 kJ/mol for the αTh-PPACK complex. Free αTh behaves differently, which explains the increased rigidity upon PPACK binding to αTh. These data suggest that αTh becomes more compact upon binding to PPACK, which is in consistent with our chemical denaturation data (Fig. 3), as estimated from the *m* unfolding parameter. The fact that these two unrelated approaches yield similar results validates these conclusions, providing evidences for understanding the structural and thermodynamics consequences of PPACK binding to αTh.

**Table 2:** Interaction energies assessed by MD for both thrombin forms.

	*Interaction Energies (kJ/mol)*	
	*Intramolecular*	*Solvent*
Free αTh	13,521.99±229.79	−17,808.90±430.83
αTh-PPACK	12,994.99±209.82	−16,678.05±373.92

## Discussion

Thrombin is a key enzyme in the coagulation cascade with multiple allosteric behaviors. αTh activity can be regulated, both by activation and inhibitory mechanisms, by a large class of small and macromolecules, such as substrates, inhibitors, glycosaminoglycans and ions [1], [3]. A detailed knowledge of the precise regulatory mechanism and ligand interaction with the active site is highly desirable because it may support the optimization of lead compounds in the design of direct αTh inhibitors and therefore in the control of thrombosis and hemostasis [45], [46]. PPACK is a well-known peptide used in covalent inhibition of αTh, and the first crystal structure of αTh was in a complex with PPACK [1], [47]. Since then no comprehensive structural or thermodynamic solution studies have been conducted with αTh. Several aspects of αTh structure and regulation have long been addressed with the crystallographic information gathered to date, such as interaction with hirugen, heparin [48], and sodium ions [3], among others compounds targeting anion-binding exosites I and II. However, despite the large amount of structural information accumulated to date, no high resolution solution structure has been made available to date. The only available information in solution reveals that two regions outside the active site undergo conformational changes leading to exclusion from the bulk solvent upon ligand binding [38], and the NMR assignment and monitoring in changes in αTh amide backbone due to modifications in the environment, conformation and/or dynamic of aminoacids by the use of two-dimensional heteronuclear correlation spectra (HSQC) [49].

Crystallization of αTh in the absence of inhibitors is limited by its autoproteolytic cleavage in exosite I at R77a [1], [3], which can be overcome by some methods [4]. Crystal structures obtained with inactive mutants are similar to wild-type, and in some cases the active site is occupied by a symmetry-related enzyme [2]. A comparative analysis of the available structures deposited in the PDB to date shows the large similarity between them, with an overall Cα RMSD of about 1 Å or less disregarding variables such as ligands, construction/mutant, pH, and solvent conditions [50]. This limited variability may be attributed to the inherent lack of structural diversity, discret ligand influence over αTh structure or even the limitations of the crystallographic method due to the solid, crystal phase condition in which the structure is solved, and also due to conformational restriction imposed by crystal packing with symmetry related αTh neighbors as previously suggested [21].

Our SAXS results indicate that both apo and PPACK-bound forms of αTh share a similar overall shape in solution. In fact, crystallographic structures of wild-type αTh in complex with PPACK (PDB entries 1PPB, 1SHH, 1SFQ; [1], [3]), in the free form (PDB entry 2AFQ [4]) or αTh mutants in the absence of ligands (PDB entries 2GP9, 1SGI, 1SG8; [3], [51] share large similarity among them (RMSD for Cα<0.8 Å), and their local differences in some regions are not detectable by SAXS measurement due to the inherent limited resolution. In opposition, circular dichroism spectra indicates that αTh is a highly variable protein, with a responsive structure upon ligand recognition and solution conditions such as pH, cation, heparin and other ligands [52]–[54]. In fact, αTh in solution display a dissimilar conformation compared to its crystal structure, as revealed by molecular dynamic simulation [21].

Despite the similarity in the overall three-dimensional solution structure of apo and holo αTh as assessed by SAXS (Fig. 1), other approaches revealed remarkable differences between them, such as changes in secondary structure as judged by CD (Fig. 2A) accompanying structural rearrangement, leading to the increase in thermodynamic stability as measured by heat and GdmCl induced unfolding (Fig. 2 and 3), and the drift in some loops from initial crystallographic conformations after MD simulation (Fig. 6).

Moreover, we have observed a complex modulation of αTh upon interaction with PPACK. In addition to the gain in stability against heat and GdmCl denaturation, an equilibrium intermediate between native and denaturant-induced unfolded αTh is populated. The chemical denaturation pathway of αTh, as shown here using GdmCl as denaturant, reveals the accumulation of an intermediate conformational state with increased catalytic activity, which has been previously shown by our group in urea-induced denaturation assays [8].

In the kinetic refolding/unfolding process a kinetic intermediate is also observed, which closely correlates with the equilibrium intermediate as shown in the *Chevron* plot (Fig. 4F). A thermodynamic change in protein stability does not rise solely from punctual interaction between the ligand (i.e., PPACK) and the enzyme (αTh). Instead, changes in intramolecular interaction are likely to take place and, consequently, conformational changes in both secondary and tertiary structure may occur upon ligand binding.

Interestingly, stopped-flow measurements demonstrated that both αTh forms behaves similarly in the kinetic refolding step, and differing in the kinetic unfolding step. We speculate that these differences in unfolding kinetics arise from differences in the consolidated, folded conformation of apo and holo αTh, indicating that PPACK binding leads αTh to a dissimilar conformational state from apo αTh, also supported by the changes in overall secondary structure content (Fig. 2A). These conclusions are not conflicting with our SAXS data, since scattering is a low-resolution technique (in this case, resolution is of about 2π/q_max_ = 30 Å), and therefore cannot provide details of secondary structure elements, giving insight only on particle properties such as Rg and Dmax.

MD techniques have been used to provide insights into the molecular basis of interaction between αTh and ligands such as DNA aptamer [55], suramin [21], heparin, thrombomodulin and Factor XIII Activation Peptide [56]. In fact, MD coupled with thermodynamic measurements from solution provides a direct access to novel aspects of protein interaction, in the energetic of binding and conformational changes upon recognition.

At the atomic scale, MD simulations also agree with the hypothesis of distinct conformational states between the two αTh forms, suggesting that PPACK binding is able to promote a reorientation of enzyme loops, mainly those surrounding the enzyme active site, promoting an increase in the overall protein rigidity and a lowering in its exposure to solvent, thus resulting in increased thermo stability of the enzyme. No major modifications in Rg and secondary structure elements were observed in the time scale of the simulations performed.

Our data provide evidences that αTh and αTh-PPACK display dissimilar conformations, especially when compared to the crystallographic free (both S195A and R77aA mutants and wild-type forms) and PPACK-bound forms of αTh. In addition, we show an intermediate state of αTh, which is found both in equilibrium and in the kinetic refolding/unfolding pathways. This novel intermediate species of αTh correlates with increased activity [8], and it is likely to possess a less rigid conformation compared to both αTh and αTh-PPACK due to the elevated concentration of denaturant when it is accumulated in equilibrium, which in the end suggests an inverse correlation between αTh rigidity and activity.

The design of drugs based on high-resolution structures of protein has a successful history in therapeutic classes such as in the case of HIV, nuclear receptors and NSAID. Most active site direct inhibitors were developed based on the described αTh-PPACK complex [5], [57]. Despite of the wealth of structural information since the elucidation of the αTh crystal structure [1], [3], approval of direct thrombin inhibitors for clinical use is very recent. The lack of a close similarity between αTh structure in crystal and solution phases may be a primary cause of delay in drug development in this field, as evidenced by the development of such inhibitors based on classical medicinal chemistry [5]. αTh was considered to be a rigid molecule with restricted conformational change [5], [58]. In contrast, we have shown a highly dynamic enzyme with a multiplicity of conformations, a common feature in biological systems with seminal importance in drug design [59].

Crystallography has long been a valuable tool in the understanding of structural principles of molecular recognition and function. We believe that the combined use of crystallographic methods as currently used in structural genomic and molecular dynamics database initiatives [60], [61], with solution studies by means of molecular dynamic simulation and the advance in the NMR characterization of αTh [49], will provide further advances in the understanding of protein function and the structure-activity relationship of αTh.
